# General anaesthetics reduce acute lymphoblastic leukaemia malignancies *in vitro* and *in vivo* *via* CXCR4 and osteopontin mediated mechanisms

**DOI:** 10.12688/f1000research.125877.3

**Published:** 2025-10-09

**Authors:** Cui Jiang, Sara Gonzalez-Anton, Xiaomeng Li, Emma Mi, Lingzhi Wu, Hailin Zhao, Ge Zhang, Aiping Lu, Cristina Lo Celso, Daqing Ma

**Affiliations:** 1 Division of Anaesthetics, Pain Medicine and Intensive Care, Division of Surgery, Department of Surgery and Cancer, Faculty of Medicine, Imperial College London, London, SW10 9NH, UK; 2 Department of Life Sciences, Imperial College London, South Kensington Campus, Imperial College London, London, SW7 2AZ, UK; 3 Haematopoietic Stem Cell Laboratory, The Francis Crick Institute, London, NW1 1AT, UK; 4 Lo Celso Laboratory, The Francis Crick Institute, London, UK; 5 School of Chinese Medicine, Hong Kong Baptist University, Hong Kong, China

**Keywords:** General anaesthetics, Propofol, Sevoflurane, Acute lymphoblastic leukaemia, Migration, Homing

## Abstract

**Background:**

Acute lymphoblastic leukaemia (ALL) is a common type of cancer in children. General anaesthetics are often used on patients undergoing painful procedures during ALL treatments but their effects on ALL malignancy remain unknown. Herein, we aim to study the effect of propofol and sevoflurane on the migration, homing and chemoresistance of ALL cells.

**Methods:**

NALM-6 and Reh cells were treated with propofol (5 and 10 μg/ml) or sevoflurane (3.6%) *in vitro* for six hours. Then, cells were harvested for adhesion assay and migration assay *in vitro*. In *in vivo* experiments, GFP-NALM-6 cells were pre-treated with propofol (10 μg/ml) or sevoflurane (3.6%) for six hours. Then, cells were injected intravenously to C57BL/6 female mice followed by intravital microscopy. For chemoresistance study, cells were treated with rising concentrations of Ara-c (0.05-50 nM) plus 10μg/ml of propofol or Ara-C plus 3.6% of sevoflurane for 4 hours, followed by the assessment of cell viability via CCK-8 assay and detection of autophagy via flow cytometry.

**Results:**

Both anaesthetics reduced *in vivo* migration and *in vivo* homing as exemplified by 1) the reduction in the number of cells entering the bone marrow and 2) the disturbance in homing location in relation to endosteal surface. Our results indicated that general anaesthetics reduced the surface CXCR4 expression and the adhesion of leukaemia cells to thrombin cleaved osteopontin (OPN) was reduced. Those changes might result in the alterations in migration and homing. In addition, both anaesthetics sensitised ALL cells to Ara-c possibly through CXCR4 mediated mechanisms. Propofol but not sevoflurane enhanced chemo-related cell death via inducing cytotoxic autophagy.

**Conclusion:**

Together, our data suggest that both propofol and sevoflurane could reduce ALL migration, and homing *in vivo* and *in vitro* via CXCR4 and OPN mediated mechanisms. Both anaesthetics could sensitise ALL cells to chemotherapy possibly via CXCR4 mediated mechanisms.

## Background

Leukaemia is the most common cancer in children, accounting for approximately one third of all childhood cancers, and the vast majority of cases (78%) are acute lymphoblastic leukaemia (ALL). ALL is the main cause of death before the age of 20 in the young with cancer. ^1^ During ALL treatments, patients repeatedly receive painful and anxiety-inducing procedures, including bone marrow biopsy, bone marrow harvest, insertion of central lines, and intrathecal chemotherapy. Young patients generally cannot tolerate these procedures under local anaesthesia alone and normally require general anaesthesia. Additionally, general anaesthesia is often required to facilitate radiotherapy procedures excluding diagnostic radiographical procedures, to ensure immobilization in young children. Therefore, paediatric patients frequently receive anaesthetics when invasive procedures are essential for diagnosis, treatment, and disease monitoring throughout treatment cycles from the induction phase to the surveillance phase, thus providing several opportunities for anaesthetics to have potential effects (if any) on leukaemia cells. ^2^^,^^3^

It has been long assumed that the behavioural and systematic effects of general anaesthetics are entirely reversible and that the central nervous system (CNS) and whole body completely recover once the anaesthetic agent is eliminated from the body. However, an increasing number of studies have shown that anaesthetics can cause long lasting effects. For example, our group firstly found that xenon (an inhalational general anaesthetic) protects the kidney against ischemia-refusion injury by upregulating hypoxia-inducible factor-1 *α* (HIF-1 *α*). ^4^ Following this discovery, other inhalational general anaesthetics like isoflurane have been shown to enhance cancer malignancy by enhancing angiogenesis, migration, invasion, and proliferation *in vitro.* ^5^^,^^6^ In line with *in vitro* findings, recent retrospective clinical data have shown an association between inhalational general anaesthesia and reduced long-term survival in cancer patients undergoing elective surgery. ^7^ Taken together, these evidence suggest that anaesthetics used during procedures may affect cancer outcomes; however, this warrants further investigation.

Migration, homing, and subsequent adhesion of leukaemia cells to a specific niche in the bone marrow are vital in leukaemogenesis, propagation of the disease, and chemoresistance. The chemokine receptor C-X-C chemokine receptor type 4 (CXCR4) and its ligand stromal-cell-derived factor 1 (SDF-1, also known as CXCL12) mediates the homing and migration process in ALL. ^8^ Sipkins *et al*. demonstrated that B-ALL cells selectively homed to adhesion molecule E-selectin and SDF-1-expressing vessels *via* a CXCR4-dependent process. Disruption of the interaction between SDF-1 and CXCR4 inhibited the homing and migration process. ^8^ Osteopontin (OPN) is an adhesive glycoprotein mainly secreted by osteoblasts and serves complex functions in bone marrow. Several isoforms of OPN exist in bone marrow due to cleavage by thrombin ^9^ and are associated with different functions. In particular, thrombin-cleaved OPN could act as an adhesion molecule for leukaemia cells in the bone marrow. ^10^

We hypothesized that general anaesthetics may affect the migration, homing and chemoresistance of ALL cells by impacting CXCR4 and OPN-mediated mechanisms. The present study, therefore, aims to investigate the impact of two commonly used general anaesthetics (propofol and sevoflurane) on leukaemia cell homing, migration and chemoresistance by using two well-established ALL cell lines (NALM-6 and Reh) *in vitro* and an engineered GFP-NALM-6 cell line *in vivo.*

## Methods

### Cell culture

Human authenticated leukaemia NALM-6 cells, RRID:CVCL_0092 and Reh Cells, RRID: CVCL_L803 were cultured in T175 tissue culture flasks (VWR, Leicestershire, UK). They were maintained at 37°C in humidified air balanced with 5% CO _2_ in RPMI 1640 medium (GIBCO, Invitrogen, Paisley, UK) supplemented with 10% heat-inactivated fetal calf serum (Thermo Scientific, Epsom, UK), 2 mM L-glutamine and 100 U ml ^-1^ penicillin-streptomycin (Invitrogen). Culture medium was replaced every day.

### Sevoflurane exposure

Before gas exposure, NALM-6 cells were cultured at 1 × 10 ^6^ per ml density on 30 mm ^2^ Petri dishes (VWR, Leicestershire, UK), or 24-well plates with a seeding density of 5 × 10 ^4^ per ml. Cells were used 12 hours after seeding. Cells were placed in 1.5 L purpose-built airtight, temperature-controlled chambers equipped with inlet and outlet valves and an internal electric fan, which was used to provide a continuous delivery and mixture of gases. The chamber was connected to calibrated flow meters and an in-line vaporizer was used to deliver the desired composition (Datex gas monitor, Helsinki, Finland) of sevoflurane (3.6%, MAC 2.0) (Abbott Laboratories, Maidenhead, UK) in 21% oxygen and 5% CO _2_ balanced with nitrogen (BOC, Guildford, UK). The chamber was pre-flushed with the aforementioned gas mixture to ensure that a stable gas composition was achieved, and a closed system was established to prevent leakage. Gas treatment was given at the desired sevoflurane concentration for two, four, and six hours at 37°C. At the end of treatment, cells were harvested for further analysis. Cells used as the naïve control group were placed in an identical gas chamber containing 21% oxygen and 5% CO _2_ balanced with nitrogen at 37°C. For recovery experiments, cells were supplied with fresh media and were returned to a standard incubator containing humidified air and 5% CO _2_ at 37°C for further analysis at 24 hours post gas exposure.

### Propofol treatment

A clinical formulation of propofol was used. It was dissolved in 10% intralipid (Astra-Zeneca, London, UK). On the day of the experiment, the stock solution of propofol was diluted with medium to the desired concentrations (5 and 10 μg/ml, equivalent to 28 and 56 μM, respectively). For the intralipid control (vehicle control), 10% intralipid was added to the cell medium to recreate the amount of intralipid in the highest (10 μg/ml) dose of propofol being used. Propofol-supplemented medium was added to the cell cultures for six hours. At the end of treatment, cells were harvested for further analysis. Cells used as the naïve control group were treated with identical medium with no propofol or intralipid. For recovery experiments, propofol medium was replaced with fresh medium and cells were returned to a standard incubator containing humidified air and 5% CO _2_ at 37°C for further analysis at 24 hours post propofol treatment.

### Chemotherapy treatment

A typical anti-mitotic chemotherapeutic agent for leukaemia, cytosine arabinoside (Ara-C), was used. For general anaesthetics treatment, 0.5-50 μM of Ara-C (Sigma-Aldrich, UK) and general anaesthetics (sevoflurane 3.6% and propofol 10 μg/ml) were given to cells for 4 hours. After treatment, cells were processed for further analysis.

### CCK-8 assay

Cells (2 × 103) were seeded in a 96-well plate and cultured in RPMI with 5% FBS in the presence of treatment compounds. Viable cell numbers were measured by formazan formation using a Cell Counting Kit 8 (Dojindo).

### Flow cytometry

For cell surface receptor experiments, leukaemia cells were incubated with CXCR4 (eBiosciecne, 17-9999-42, APC) and CD49d (eBioscience, 12-0499-42, PE) antibodies for 30 minutes at 4°C. After incubation, cells were washed with PBS and samples were run on a flow cytometer (CyAn ADP; Beckman Coulter, US). In addition, isotype controls were used to establish background fluorescence signals including: 1) mouse IgG2a kappa isotype control eBM2a (eBioscience, 17-4724-81, APC) and 2) mouse IgG1 kappa isotype control P3.6.2.8.1 (eBioscience, 12-4714-82, PE). In order to gate cells, the scatter plot of forward scatter versus side scatter was initially gated to have *via*ble cells. Then, duplicates were excluded by plotting forward scatter height versus area. Finally, protein expression represented by mean fluoresce intensity (MFI) was recorded using a histogram. Each assay included 50,000 gated events. All samples were analysed *via* FlowJo software. For propidium iodide experiments, cells were stained with propidium iodide (e-Bioscience, 00-6990-50, PE-Texas red) and incubated in the dark at room temperature for 5 minutes. A count of 50,000 cells per sample were analysed with a flow cytometer (CyAn ADP; Beckman Coulter, US). In order to determine the cell death *via* propidium iodide, the scatter plot of forward scatter versus the PE-Texas was initially analysed in naïve control samples to determine the population of healthy cells. We determined any cells with an intensity larger than 10 ^2^ in the PE-Texas red channel to be dead cells.

### *In vitro* migration assay

*In vitro* migration assay was performed by loading 2 × 10 ^5^ pre-treated cells into the upper chamber of the migration chamber. Cells were allowed to migrate for six hours before being collected in the lower chamber. The lower chamber was supplied with 20 ng/ml SDF-1 as a chemoattractant. Finally, samples were run on a flow cytometer (CyAn ADP; Beckman Coulter, US) with Accu check counting beads (Thermo Fisher, PCB 100, UK) to determine absolute cell counts in each sample.

### *In vitro* adhesion assay

NALM-6 cells were pre-treated with propofol and sevoflurane with respective concentrations. Recombinant human OPN (R&D systems, 1433-OP-050/CF) was thrombin-cleaved by incubating 24 μg recombinant full length human OPN in 20 mM Tris-HCL (pH7.6), 80 mM NaCl, 2 mM CaCl _2_, and 0.1 unit thrombin (Merck, 10602400001) for ten minutes at 37 degrees. Then, full length OPN and thrombin-cleaved OPN were coated at 20 μg/ml the night before experiment. Then, 5 × 10 ^5^ cells of NALM-6 cells were labelled with calcein-AM stock solution from the Vybrant cell adhesion assay kit (ThermoFisher, UK). Pre-labelled NALM-6 cells were allowed to adhere for two hours under 37°C. Finally, fluorescence readings were read at 494 nm. The percentage of adhesion was determined by dividing the corrected (background subtracted) fluorescence of adherent cells by the total corrected fluorescence of cells added to each microplate well and multiplying by 100%.

### Mouse study

All animal work was in accordance with the animal ethics committee (AWERB) at Imperial College London, UK and the UK Home Office regulations (ASPA, 1986). C57BL/6 female mice were purchased from Charles rivers UK Ltd with minimum age >6 weeks with weight around 20-22 g. All animals were allowed for 7 days of acclimation before procedures.

Prior to procedures, animals were randomly allocated into three groups: 1) naïve control group, 2) propofol treated group and 3) sevoflurane treated group. In naïve control group, animals were given one million untreated GFP-NALM-6 cells through tail vein injection followed by intravital microscopy. In propofol and sevoflurane treated group, animals were given one million pre-treated GFP-NALM-6 cells (pre-treated with 10 μg/ml propofol and 3.6% sevoflurane for six hours *in vitro*, respectively) followed by intravital microscopy. On the day of experiments, we would normally do three intravital microscopy procedures in the order of 1) naïve control group, 2) propofol treated group and 3) sevoflurane treated group to minimise potential confounders. All members involved in the mouse study were aware of group allocations at all stages.

### GFP-expressing virus transduction

Initially, 5 × 10 ^4^ of NALM-6 cells were seeded in a 24-well plate. After 12 hours, TransDux and TransDUX MAX enhancer (System Biosciences, Palo Alto, USA) were mixed and added to culture at a concentration of 1X. Then, pre-packed GFP lentivirus (System Biosciences, Palo Alto, USA) was added to NALM-6 cells at a multiplicity of infection (MOI) of 20. Cells were incubated in 5% CO _2_ at 37°C for 72 hours. Finally, GFP-expressing NALM-6 cells were sorted using FACS Aria-II cell sorter (BD, USA), before seeding in a T175 flask with fresh medium.

### Intravital microscopy

Intravital microscopy is carried out after injections of GFP expressing cells. Intravital microscopy was performed using a Leica SP5 and a Zeiss LSM 780 upright confocal microscopes, both with a motorized stage. ^11^ The SP5 was fitted with the following lasers: Argon, 546, 633 and a tunable infrared multiphoton laser (Spectraphysics Mai Tai 690-1020). The Zeiss LSM 780 was fitted with the following lasers: Argon, 561, 633 and a tuneable infrared multiphoton laser (Spectraphysics Mai Tai DeepSee 690-1040). The signal was visualized with a Leica HCX IRAPO L ×25 water immersion lens (0.95 N.A) and a W Plan-Apochromat ×20 DIC water immersion lens (1.0 N.A), respectively. Collagen bone second harmonic generation signal and GFP and CFP signals were generated through excitation at 840 and 870 nm and detected with external detectors. Internal detectors were used to collect DsRed and Cy5 signals (and on some occasions, GFP). Anaesthesia was induced in mice with 4% isoflurane mixed with pure oxygen. This was gradually reduced to approximately 1% as anaesthesia stabilised. Animals were randomly assigned to each group (naïve control, propofol treated, and sevoflurane treated).

Surgery to attach the headpiece was then performed as described below.

1) Sterile forceps and scissors were used to carefully remove the central portion of the scalp to expose the calvarium area to be imaged. Then, a small incision was made at the back of the head between the ears by lifting the skin up with the forceps. While holding the skin up, scissors were slid under the skin and gently cut along the outside of the desired imaging area.
2) An adequate amount of dental cement was mixed in a weigh boat until it became a paste and it was quickly applied to the bottom surface of the headpiece that will attach to the skull.
3) Before the cement set, headpiece was placed onto the skull of the mouse, making sure not to get any dental cement on the imaging area. Then, it was important to wait for it to set.
4) The headpiece was attached to the holder and secured in place using the screw, ensuring that the grooves fit within the holder notches.

Large three-dimensional ‘tile scans’ of the entire BM cavity space were acquired by stitching adjacent, high-resolution z-stack images using a surgically implanted imaging window that ensures steady positioning of mice on the microscope. Time-lapse datasets were acquired by focusing on one area of BM. One image was taken every three minutes for a total of 22 images. Then, all 22 images were combined together to produce a movie (time-lapse). The calvarium has been demonstrated to be equivalent to the long bones such as the femur with regards to haematopoietic stem cell frequency, function, and localization, and is the only BM compartment that allows longitudinal imaging through minimally invasive surgery. Blood vessels were highlighted by I.V. injection of 50 μl of 8 mg/ml 500 kDa Cy5-Dextran (Nanocs, MA). After the intravital microscopy, all mice were euthanised *via* cervical dislocation.

### Image quantification

Microscopy data was processed using multiple platforms. Tile scans were stitched using ZEN black, RRID:SCR_018163 (Zeiss, Germany) software. Raw data were visualised and processed using Fiji/Image J, RRID:SCR_00285 (National Institutes of Health, Maryland, USA). Cell tracking was performed using FIJI plugin MTrackJ. For accuracy in cell tracking data, videos were registered when required before using four-dimensional data protocols implemented in Fiji. Three-dimensional data rendering and measurement of cell distances were performed in Volocity, RRID:SCR_002668 (Perkin Elmer, MA, USA).

### Statistical analysis

The sample size required for the experiments was estimated based on the results of preliminary data. Statistical differences between the means of two data groups were determined using two-tailed unpaired Student’s t-test, and p values<0.05 were considered statistically significant. Multiple group comparisons were performed using ANOVA with a Kruskal-Wallis nonparametric or Bonferroni’s *post hoc* test for comparisons GraphPad Prism 7.0, RRID:SCR_002798. A p value<0.05 was considered statistically significant.

## Results

### General anaesthetics reduce the migration of leukaemia cells *in vitro* and *in vivo*

**Figure 1.**
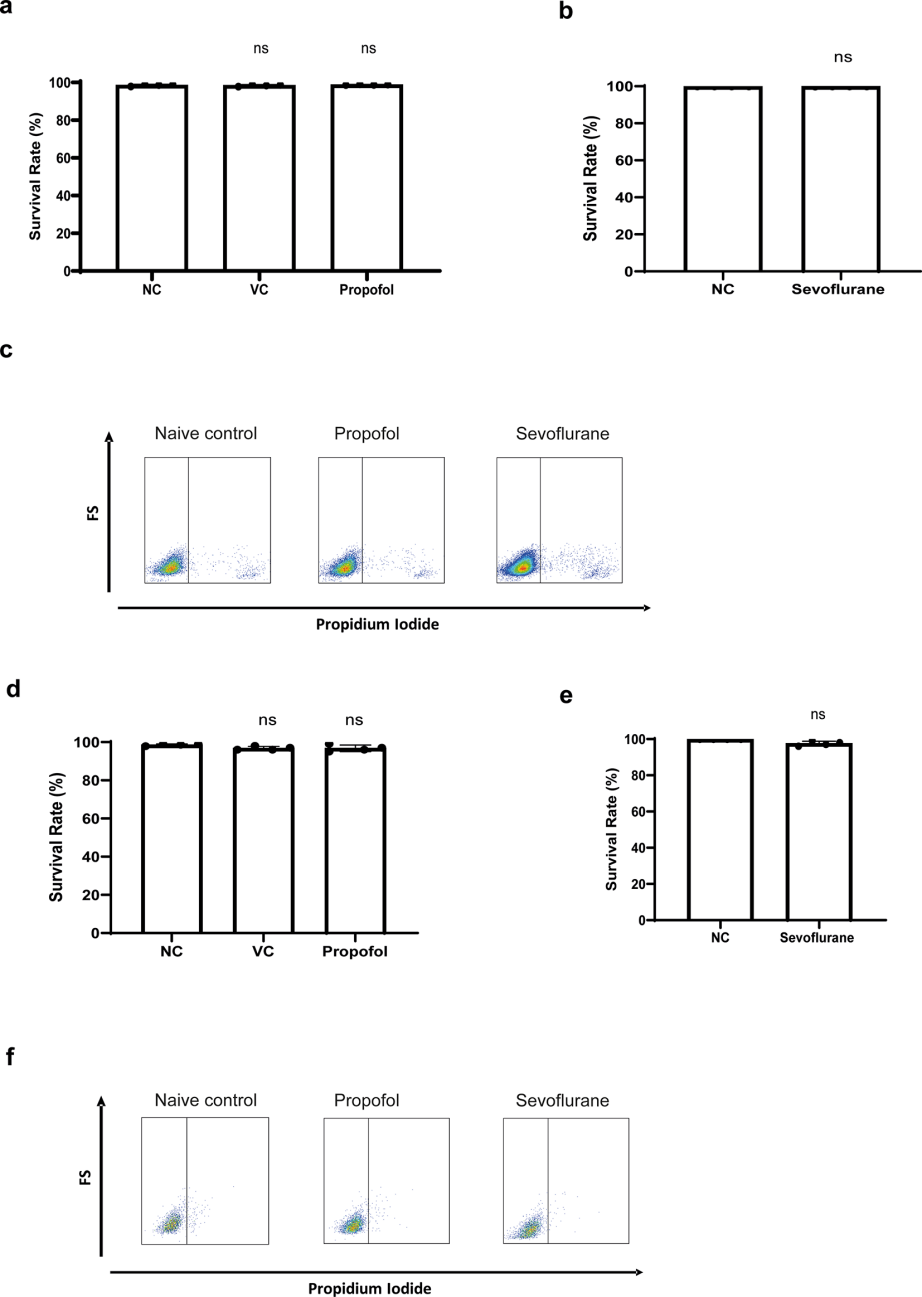
General anaesthetics do not affect cell *via*bility of NALM-6 and Reh cells. a-b) NALM-6 cells were treated with intralipid, 10 μg/ml propofol and 3.6% sevoflurane for six hours. Cell *via*bility was detected using through flow cytometry by propidium iodide-based assay. Data are illustrated as mean±sd (n=4). NC: Naïve control. VC: Vehicle control (Intralipid). d-e) Reh cells were treated with 10 μg/ml propofol and 3.6% sevoflurane for six hours. Cell *via*bility was detected through flow cytometry by propidium iodide-based assay. Data are illustrated as mean±sd (n=4). c and f) Flow plot images shown here are representative images selected from 4 repeats.

Before assessing their effects on cell migration, cell toxicity of propofol and sevoflurane were assessed *via* propidium iodide staining. No cell death was detected in NLAM-6 and Reh cells after exposure to propofol and sevoflurane ( Figure 1a- f).

**Figure 2.**
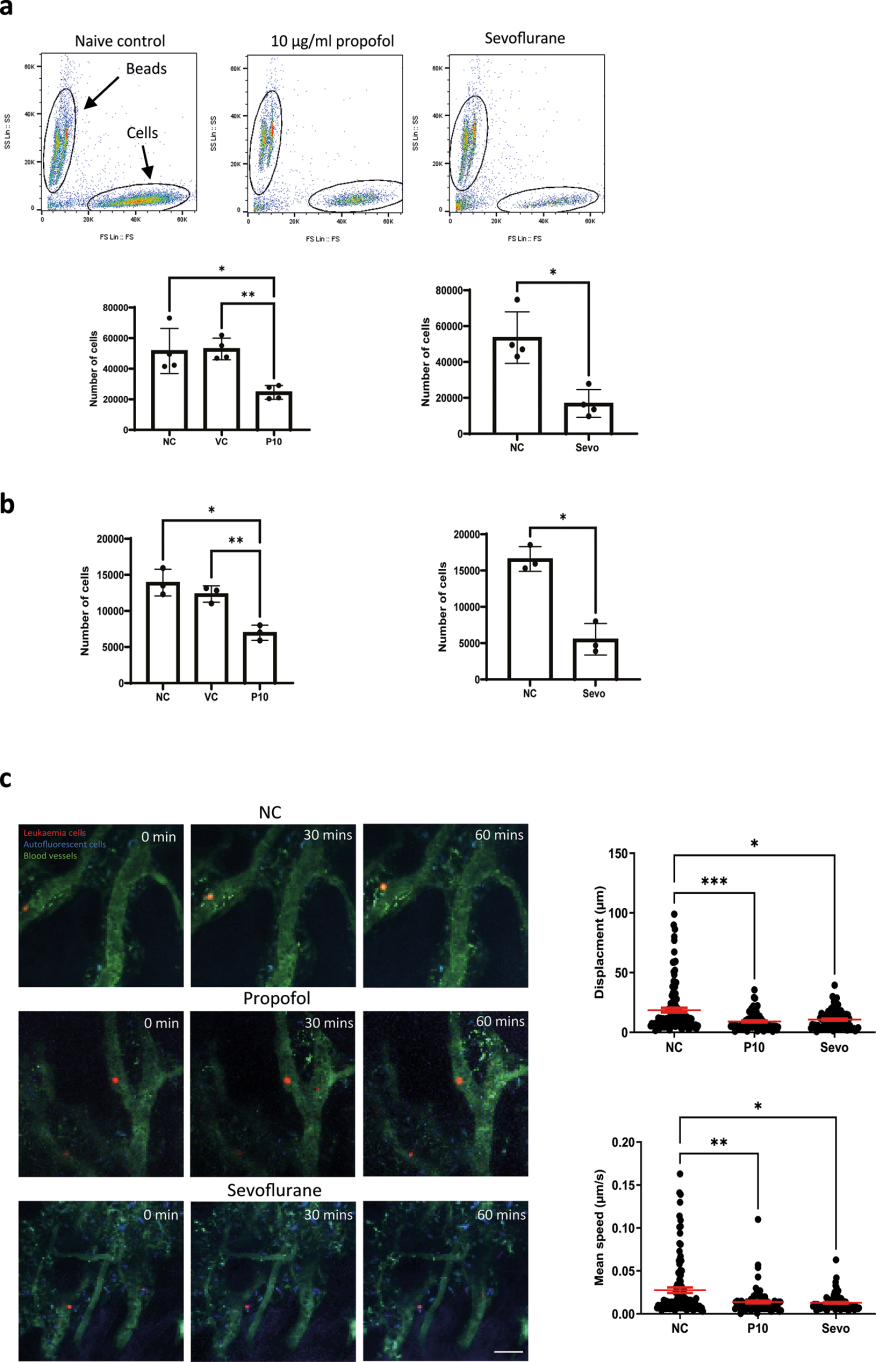
General anaesthetics reduce cell migration *in vitro* and *in vivo.* a) NALM-6 cells were initially treated with intralipid (VC), 10 μg/ml propofol and 3.6% sevoflurane for six hours. Then, treated cells were loaded into the migration chamber with 1 nM SDF-1 in the lower chamber as the chemoattractant. Cells were allowed to pass through migration chambers for six hours. Cells in the lower chamber were collected and counted in a flow cytometer. Data are shown as mean±sd (n=4). NC: naïve control, VC: intralipid, P10: 10 μg/ml propofol, and Sevo: 3.6% sevoflurane. *p<0.05, **p<0.01, b) Reh cells were initially treated with intralipid (VC), 10 μg/ml propofol and 3.6% sevoflurane for six hours. Then, treated cells were loaded into the migration chamber with 1 nM SDF-1 in the lower chamber as the chemoattractant. Cells were allowed to pass through migration chambers for six hours. Cells in the lower chamber were collected and counted in a flow cytometer. Data are shown as mean±sd (n=4). NC: naïve control, VC: intralipid, P10: 10 μg/ml propofol, and Sevo: 3.6% sevoflurane. *p<0.05, **p<0.01 c) Intravital confocal calvarium imaging of GFP-transduced NLAM-6 cells (red) was performed after injection of GFP-NALM-6 cells into C57BL/6 mice IV. GFP-NALM-6 cells were pre-treated with either 10 μg/ml of propofol or 3.6% sevoflurane overnight *in vitro* prior to injections. Respective time-lapses were shown following IV injection of tritc-dextran to identify blood vessels (green); non leukaemia cells with high autofluorescent signals (blue) were recorded as well to improve the identification of target cells. Scale bar: 50 μm. Each position was imaged at three-minute intervals for 66 minutes. We only investigated the migration of cells in bone marrow. Cells in blood vessels were excluded from our investigations. Top row: Mouse injected with untreated cells (red). NC: naïve control. Middle row: Cells were pre-treated with 10 μg/ml of propofol overnight *in vitro* prior to injection. Bottom row: Cells were pre-treated with 3.6% of sevoflurane prior to injection. Displacement of each cell captured in all time-lapses was recorded. Data are pooled from 3 naïve control mice (N=3, 113 cells), 4 mice injected with propofol treated cells (n=4, 86 cells) and 4 mice injected with sevoflurane treated cells (N=4, 88 cells). Data are illustrated as mean±sd. For displacement data, *p<0.05, ***p<0.001. Data are analysed by one-way non-parametric ANOVA test (Kruskal-Wallis test). Propofol: 10 μg/ml of propofol and Sevoflurane: 3.6% of sevoflurane. Mean speed of each cell captured in time-lapses was recorded. Data is pooled from 3 naïve control mice (N=3, 113 cells), 4 propofol pre-treated mice (N=4, 86 cells) and 4 sevoflurane treated mice (N=4, 88 cells). Data are illustrated as mean±sd. *p<0.05, **p<0.01. Data is analysed by one-way non-parametric ANOVA test (Kruskal-Wallis test). Propofol: cells were pre-treated with 10 μg/ml of propofol for six hours and Sevoflurane: cells were pre-treated with 3.6% of sevoflurane for six hours.

We investigated whether the migration of leukaemia cells was affected by general anaesthetics. An *in vitro* migration chamber was set up where 2 × 10 ^5^ of NLAM-6 and Reh (pre-treated with propofol or sevoflurane for six hours) were loaded into the upper chamber and allowed to migrate for six hours to the lower chamber where 1 nM of SDF-1, a CXCR4 ligand, was applied. We observed that 10 μg/ml (56 μM) of propofol led to a significant reduction in the number of cells migrating to the lower chamber (almost a 50% decrease) ( Figure 2a-b). Similarly, sevoflurane, significantly reduced cell migration to the lower chamber ( Figure 2a-b). Intralipid (vehicle control) had no effect on cell migration.

In order to validate our *in vitro* migration results, we set up a mouse model to study migration using intravital microscopy. ^12^ In our model, we used immune-competent C57BL-6 mice instead of SCID mice for homing and migration experiments as it is a well-established model for non-maintenance cancer studies. ^13^ GFP-NALM-6 cells (pre-treated with 10 μg/ml propofol or 3.6% sevoflurane for six hours) were injected intravenously. Then, intravital microscopy was performed. Only cells present in all time lapse images were tracked and included in our displacement analysis. In addition, cells inside blood vessels were not included in analysis. Displacements were calculated by adding all displacements in each time interval. The mean speed was calculated by averaging all mean speed recorded in each time interval. Our results showed that general anaesthetic pre-treated cells appeared to achieve smaller displacements compared to naïve cells, and their mean migration speed was also significantly reduced ( Figure 2c). It was very obvious that after propofol and sevoflurane exposure, the number of fast-moving cells was significantly reduced ( Figure 2c).

### General anaesthetics reduce the homing of NALM-6 cells *in vivo*

**Figure 3.**
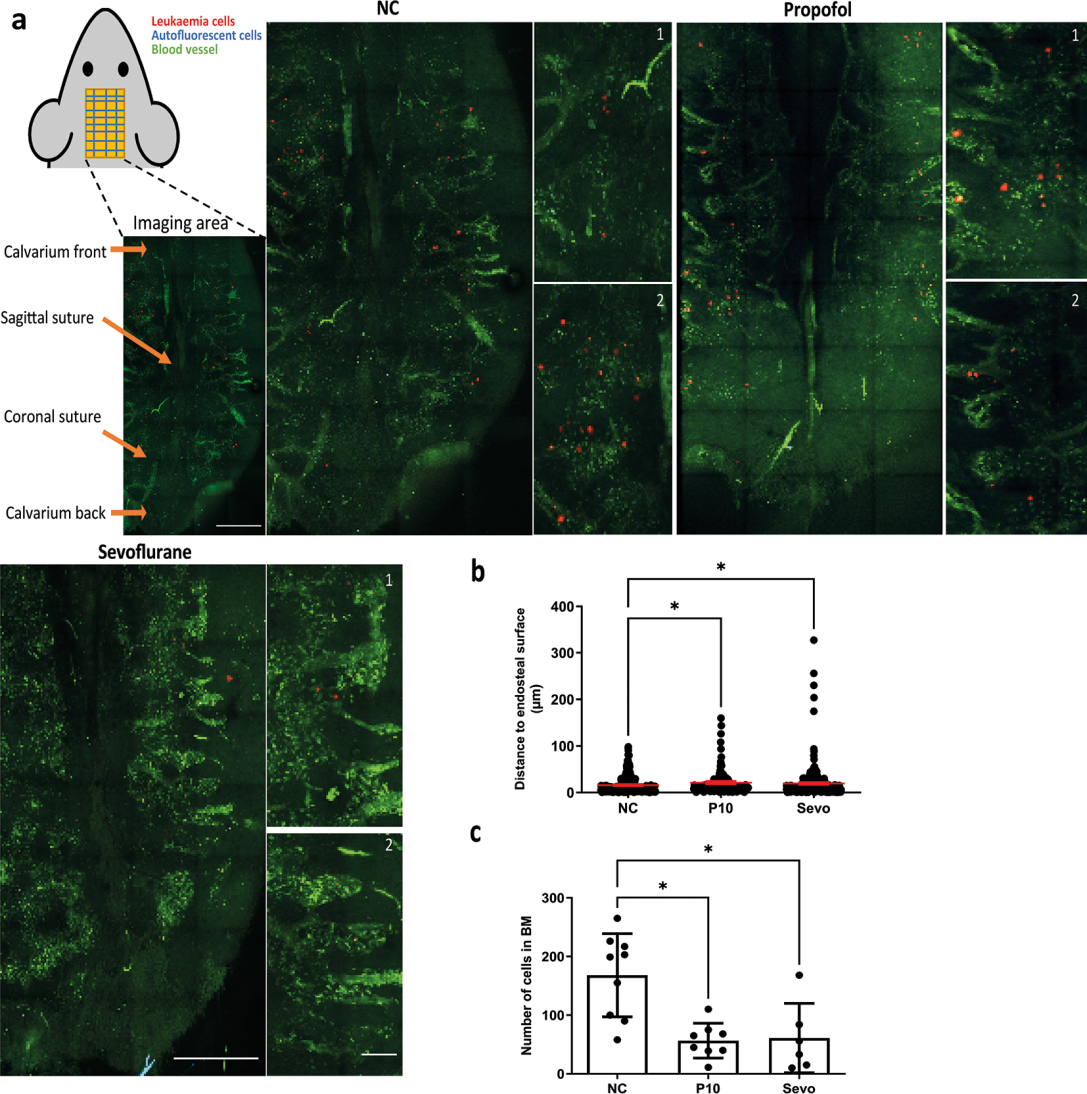
General anaesthetics affect the homing of ALL cells entering bone marrow and affect the homing location of ALL cells *in vivo.* a) Upper left: diagram showing strategy for calvarium imaging. Intravital confocal calvarium imaging of GFP-transduced NLAM-6 cells (red) was performed after injection of GFP-NALM-6 cells into C57BL/6 mice IV. GFP-NALM-6 cells were pre-treated with either 10 μg/ml of propofol or 3.6% of sevoflurane prior to injection. Representative maximum projection tile scans and corresponding high-magnification inserts were shown following IV injection of tritc-dextran to identify blood vessels (green); non leukaemia cells with high autofluorescent signals (green, spotty signal) were recorded as well to improve the identification of target cells. Higher magnification images show portions of low magnification tile scans. Scale bar: 500 μm for low magnification tile scan. 50 μm for high magnification images. b) The distance between the nearest bone surface (not shown) and each GFP-NALM-6 cell was measured. Data illustrated as mean±sd. Data are pooled from 4 naïve control mice (N=4, 279 cells), 4 mice injected with propofol treated cells (n=4, 134 cells) and 6 mice injected with sevoflurane treated cells (n=6, 366 cells). *p<0.05. Data are analysed by one-way non-parametric ANOVA test (Kruskal-Wallis test). c) We calculated the number of cells enter bone marrow space from IVM images for each treatment group. Data are illustrated as mean±sd. Data are pooled from nine naïve control mice, eight mice injected with propofol pre-treated cells and six mice injected with sevoflurane pre-treated cells. *p<0.05. Data are analysed by one-way non-parametric ANOVA test (Kruskal-Wallis test). Propofol: cells were pre-treated with 10 μg/ml of propofol for six hours, Sevoflurane: cells were pre-treated with 3.6% of sevoflurane for six hours.

After revealing that cell migration was reduced by general anaesthetics, we investigated whether the homing process was affected by anaesthetics specifically in terms of 1) homing location of cells (expressed by the distance between each cell to the nearest endosteal surface); 2) The number of cells entering bone marrow. Tile scan imaging ( Figure 3a) of large areas of mouse calvarium bone marrow was carried out and the distance between each cell and the nearest endosteal bone surface was calculated. We used this distance as an indication for homing location. Cells in the blood vessels were not included in our analysis. Results showed that the average distance between a leukaemia cell and the endosteal bone surface was increased by propofol and sevoflurane, indicating disruption of the homing process ( Figure 3b). Naïve control cells consistently move to locations approximately within 100 μm of the endosteal surface in bone marrow ( Figure 3b). Presumably, this is a discrete region near vessels demonstrated previously, ^8^ though the previous publication did not measure the distance between cells and the endosteal bone surface. After exposure to propofol or sevoflurane, a fair proportion of cells was found in locations further away from the endosteal bone surface ( Figure 3b).

We then studied whether treatment with general anaesthetics affected the number of cells entering bone marrow by counting the number of GFP+ cells from tile scans. Our results demonstrated that treatment with propofol and sevoflurane caused a significant reduction in the number of NALM-6-GFP cells entering bone marrow ( Figure 3c). After either treatment, fewer than 100 GFP-NLAM-6 cells entered the bone marrow compared to an average of nearly 200 cells entering the bone marrow for naïve control cells.

### General anaesthetics reduce the leukaemia cell surface CXCR4 expression and reduce the adhesion of leukaemia cells to thrombin-cleaved OPN *in vitro*

**Figure 4.**
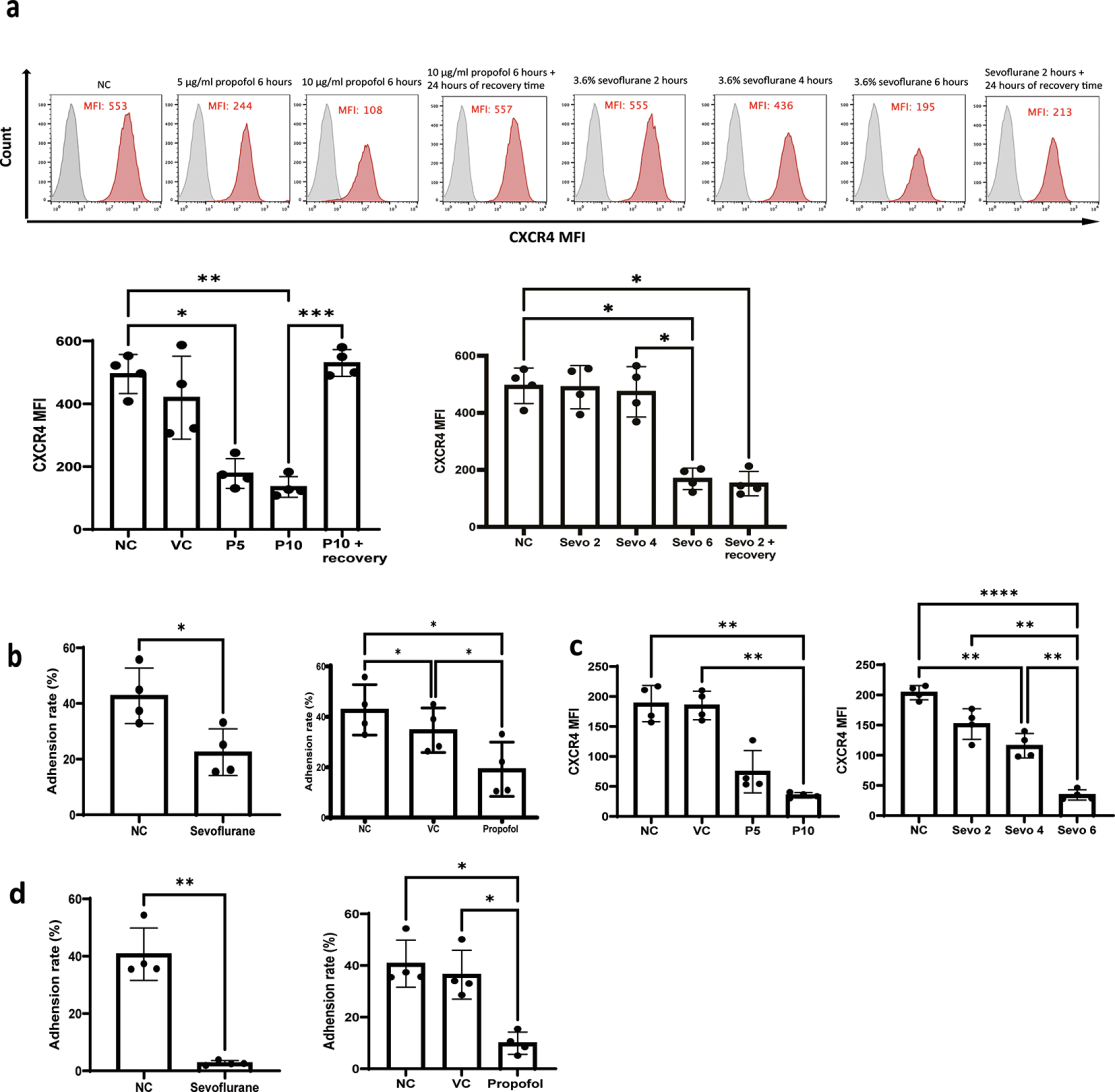
General anaesthetics reduce cell surface CXCR4 and *in vitro* adhesion to thrombin-cleaved osteopontin. a) NALM-6 cells were treated by intralipid (VC), 5 μg/ml and 10 μg/ml of propofol for six hours and 24 hours of recovery time is given for samples treated with 10 μg/ml of propofol. Or cells were treated by 3.6% of sevoflurane for two, four, and six hours, and 24 hours of recovery time is given to samples treated by initial 2 hours of exposure of sevoflurane. Following the general anaesthetics treatment and recovery time, treated cells were stained with a CXCR4 antibody and analysed by flow cytometer for mean fluorescence intensity. Data are illustrated as mean±sd (n = 4). Data are analysed by one-way ANOVA followed by Bonferroni’s *post hoc* test. For propofol statistics, *p<0.05, **p<0.01, ***p<0.001. VC: intralipid, P5: 5 μg/ml of propofol, P10: 10 μg/ml of propofol, Sevo 2: two hours sevoflurane, Sevo 4: four hours sevoflurane and Sevo 6: six hours sevoflurane. Grey histogram: isotype control; Red histogram: CXCR4. b) NALM-6 cells were either left untreated or treated with 3.6% sevoflurane for 6 hours. Alternatively, NALM-6 cells were either left untreated or treated with 10 μg/mL propofol or intralipid for 6 hours. Following treatment, cells were allowed to adhere for 2 hours to plates pre-coated with thrombin-cleaved osteopontin. Data are presented as mean±sd (n = 4). *p < 0.05. Statistical analysis was performed using one-way ANOVA followed by Bonferroni’s post hoc test. c) Reh cells were treated by intralipid (VC), 5 μg/ml and 10 μg/ml of propofol for six hours. Or cells were treated by 3.6% of sevoflurane for two, four, and six hours. Following the general anaesthetics treatment, treated cells were stained with a CXCR4 antibody and analysed by flow cytometer for mean fluorescence intensity. Data are illustrated as mean±sd (n = 4). Data are analysed by one-way ANOVA followed by Bonferroni’s *post hoc* test. **p<0.01. ****p<0.0001. VC: intralipid, P5: 5 μg/ml of propofol, P10: 10 μg/ml of propofol, Sevo 2: two hours sevoflurane, Sevo 4: four hours sevoflurane and Sevo 6: six hours sevoflurane. d) Reh cells were either left untreated or treated with 3.6% sevoflurane or 10 μg/mL propofol or intralipid for six hours. Following treatment, cells were allowed to adhere for two hours to plates pre-coated with thrombin-cleaved osteopontin. Data are presented as mean±sd (n = 4). Statistical analysis was performed using one-way ANOVA followed by Bonferroni’s *post hoc* test (*p < 0.05, **p < 0.01). VC: intralipid.

CXCR4 is crucial for migration ^14^ and homing ^8^ of leukaemia cells in the bone marrow. One study demonstrated that B-ALL cells (NALM-6 cells) home to discreet, discontinuous locations near vessels in bone marrow that express the adhesion molecules E-selectin and SDF-1. ^8^ Disruption of the interactions of the SDF-1-CXCR4 axis inhibited the homing of GFP-NALM-6 cells to these vessels. ^8^ We hypothesised that CXCR4 was one of the key factors in general anaesthetics-mediated reduction of the migration and homing of GFP-NALM-6 cells *in vivo.* In order to validate this, we treated NALM-6 and Reh cells with propofol (5 and 10 μg/ml) and sevoflurane (3.6% MAC 2) followed by flow cytometry analysis of surface CXCR4 expression. In addition, 24 hours of recovery time is given to propofol and sevoflurane treated samples to study whether the effect of general anaesthetics on CXR4 expression is long-lasting. Our results indicated that both propofol and sevoflurane significantly reduced surface CXCR4 expression ( Figure 4a and c). More specifically, 5 and 10 μg/ml of propofol (28 and 56 μM) reduced CXCR4 expression in a dose-dependent manner ( Figure 4a and c). Only six hours of sevoflurane significantly reduced CXCR4 expression in NALM-6 cells ( Figure 4a). Both 4 hours and 6 hours of sevoflurane could significantly reduce CXCR4 expression in Reh cells ( Figure 4c). These results suggest that CXCR4 might be the key behind general anaesthetics-mediated reduction of homing and migration in leukaemia cells. Interestingly, we found that propofol’s reductive effect on CXCR4 expression is not long-lasted as after 24 hours of recovery time, CXCR4 expression resumed to naive control level. However, reductive effect on CXCR4 expression is sustained for sevoflurane exposure as CXCR4 expression is consistently suppressed after 24 hours of recovery time with initial 2 hours pre-treatment of sevoflurane ( Figure 4a). These results suggest sevoflurane but not propofol may affect homing and migration of ALL cells in the long term.

In addition to CXCR4, we then speculated that the changes in the number and position of GFP-NALM-6 cells homed to the BM was due to the disruption of interactions between leukaemia cells and components of bone marrow matrix. We firstly investigated the expression of integrin α4 (CD49d), which is one of the dimers of α4β1 integrin or very late antigen 4 (VLA-4) (the other one being integrin β1), after exposure to general anaesthetics. VLA-4 is an essential mediator for engraftment of NALM-6 cells in bone marrow by anchoring NALM-6 cells to bone marrow matrix and supporting leukaemia growth. ^10^^,^^15^^,^^16^ The prime target for VLA-4 adhesion is osteopontin (OPN). Interestingly, no significant change was detected in the expression of the α4 subunit after anaesthetics exposure (Supplementary figure 1a and b). Next, we hypothesised that general anaesthetics might affect the adhesion of leukaemia cells to OPN. Given that OPN has various isoforms in bone marrow, we tested the adhesion of leukaemia cells to thrombin-cleaved OPN, which is the most dominant form of OPN in bone marrow. ^9^ Similar to a previous study, ^10^ leukaemia cells only weakly adhered to full-length OPN but they showed strong adhesion to thrombin-cleaved OPN (Supplementary figure 1c). Consistent with our hypothesis, when NALM-6 and Reh cells were pre-treated with propofol and sevoflurane, the percentage of cells adhered to thrombin-cleaved OPN was significantly reduced ( Figure 4b and d).

### General anaesthetics sensitise NALM-6 and Reh cells to a commonly used chemotherapeutic agent (Ara-C)

**Figure 5.**
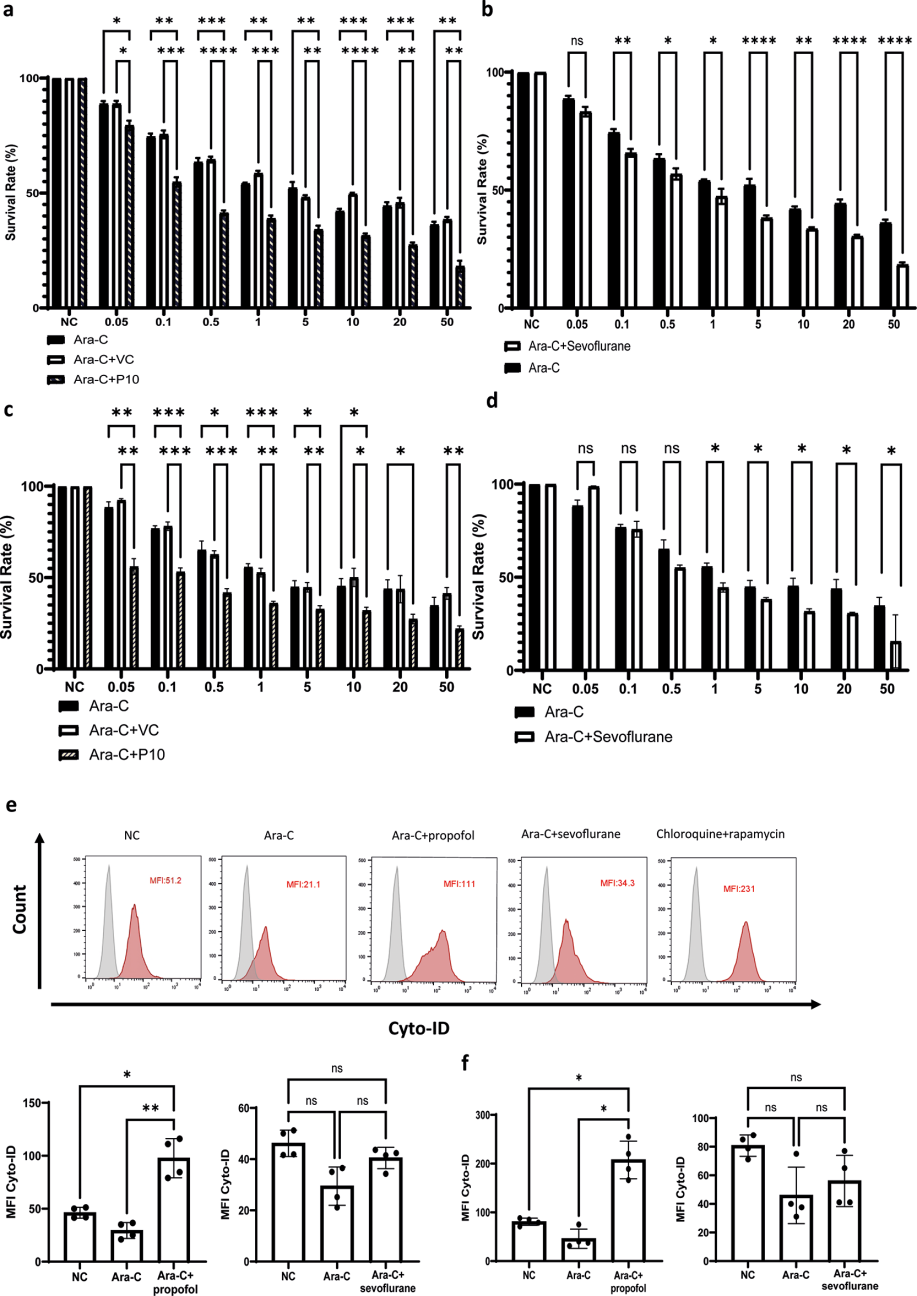
General anaesthetics enhance the chemo-sensitivity of NALM-6 and Reh cells. a and c) NALM-6 and Reh cells were treated with 10 μg/ml propofol or intralipid and Ara-C (0.05 to 50μM) for 4 hours. Cell viability analysis was carried out by CCK-8. *p<0.05, **p<0.01, ***p<0.001 and ****p<0.0001. Data is shown as mean±sd (n=4). Data is analysed by two-way ANOVA followed by Bonferroni’s post-hoc test. VC: Vehicle control, P10: 10μg/ml. b and d) NALM-6 and Reh cells were treated with 3.6% sevoflurane and Ara-C (0.05 to 50μM) for 4 hours. Cell viability analysis was carried out by CCK-8. Data is shown as mean±sd (n=4). *p<0.05, ****p<0.0001. Data is analysed by two-way ANOVA followed by Bonferroni’s post-hoc test. e-f) NALM-6 and Reh cells were treated with 0.1 μM Ara-C, Ara-C plus 10 μg/ml propofol or intralipid and Ara-C plus 3.6% sevoflurane for 4 hours followed by flow cytometry analysis of autophagy influx by Cyto ID autophagy detection kit. Data is illustrated as mean±sd (n=4). *p<0.05, **p<0.01. Data is analysed by one-way ANOVA followed by Bonferroni’s post-hoc test. Grey histogram: Isotype control, Red histogram: Cyto-ID.

Chemoresistance is the main challenge in treating leukaemia. To study whether the mechanisms we uncovered may impact chemoresistance, we treated NLAM-6 and Reh cells *in vitro* with rising concentrations of Ara-c (0.05-50 nM) plus 10 μg/ml (56 μM) of propofol or Ara-C (0.05-50 nM) plus 3.6% of sevoflurane (MAC 2.0) for 4 hours, followed by the assessment of cell viability via CCK-8 assay. Propofol increased cell death of NALM-6 and Reh cells at all concentrations of Ara-C and sevoflurane enhanced the cytotoxicity induced by Ara-C at most of its concentrations in NALM-6 cells. In Reh cells, sevoflurane enhanced cytotoxicity in higher Ara-C concentrations (i.e., 1-50 nM) ( Figure 5a- d). Our initial hypothesis was that both propofol and sevoflurane induced cytotoxic autophagy, as both agents are known autophagy inducers. ^17^ However, we found that propofol induced autophagy across NALM-6 and Reh cells but sevoflurane did not induce autophagy using flow cytometry based Cyto-ID autophagy detection kit ( Figure 5e and f). Samples treated by propofol plus Ara-C had almost double mean fluorescence intensity (MFI) in Cyto-ID than control samples, whereas Ara-C+sevoflurane treatment did not affect the MFI of Cyto-ID. Collectively, propofol likely increased the cytotoxicity of Ara-C via activating cytotoxic autophagy and enhancing cell death pathways in vitro. However, the exact mechanism by which sevoflurane triggers cell death pathways following the treatment of Ara-C still warrants further investigation.

## Discussion

Our work, for the first time, demonstrates that general anaesthetics reduce the migration and homing of ALL cells *via* CXCR4 and OPN mediated mechanisms. By using *in vitro* models and an immunocompetent mouse model, we found that both propofol and sevoflurane lead to a reduction in CXCR4 expression and disruption of adhesion to thrombin-cleaved osteopontin in leukaemia cells. These effects were associated with reprogramming of the bone marrow microenvironment to unfavourable conditions for leukaemia cells, as illustrated in the reduction of migration speed, the disruption of homing locations, and the reduction in the number of cells entering bone marrow. In addition, our results showed that both general anaesthetics sensitise ALL cells to chemotherapy possibly via CXCR4 mediated mechanisms.

The importance of CXCR4 (specifically the SDF-1-CXCR4 axis) in B-ALL homing and migration has been well documented. ^8^^,^^14^^,^^18^ Our CXCR4 expression data is novel and provides direct evidence for the effect of anaesthetics on migration, demonstrating that general anaesthetics reduce CXCR4-dependent chemotaxis in NALM-6 and Reh cells. In addition, results from recovery time experiment showed sevoflurane but not propofol could exert long-term (24 hours) effects on CXCR4 expression.

Homing is a vital process in the early stages of leukaemia development. It allows leukaemia cells to seed, multiply, and importantly, evade detection by the immune system. B-ALL cells have been demonstrated to home to unique anatomic regions in bone marrow. ^8^ It was reasonable to assume that the suppression of CXCR4 by general anaesthetics was one of the reasons for the disruption of homing in our study. Our *in vitro* data also showed that the adhesion of ALL to thrombin-cleaved osteopontin is reduced by propofol and sevoflurane. Thrombin-cleaved osteopontin is a prominent adhesion molecule in bone marrow, which regulates the binding of leukaemia to bone marrow niches required for homing. The mechanism behind the reduction in adhesion caused by general anaesthetics is not known as VLA-4 receptor expression is not affected by general anaesthetic exposure. In addition to homing disturbance, we found that the number of leukaemia cells entering the bone marrow space was reduced by general anaesthetic treatment *in vivo.* This may possibly be due to the reduction of surface CXCR4 on NALM-6 and Reh cells caused by general anaesthetics. 

The findings on Ara-C chemosensitivity following general anaesthetic treatment are interesting. It is likely that the reduction of expression of CXCR4 is related to the enhancement of chemosensitivity given some have reported the CXCR4 antagonist the chemoresistance *in vitro* in ALL cells. ^19^ In addition, X Hu et al has reported that CXCR4 may mediated autophagy signalling via SIRT1 and enhanced chemoresistance in AML cells. ^20^ It could be reasonability speculated that at least propofol enhanced cytotoxicity of Ara-C via cytotoxic autophagy mediated by CXCR4 related mechanisms. However, Sevoflurane may cause a non-autophagic form of cell death when co-administered with Ara-C as we did not detect the upregulation of autophagy signals.

Looking at clinical studies, a few retrospective studies and one meta-analysis have investigated the beneficial effect of general anaesthetics on cancer outcomes, and propofol may be associated with improved recurrence-free survival and overall survival in patients having cancer surgery. ^7^^,^^21^^–^^25^ Further clinical studies, including whether propofol is beneficial for ALL patients, are urgently needed. Ideally, anaesthetics without any effects on cancer cell malignancy or, even better, with some potential anti-cancer properties should be chosen to use in cancer patients during surgery. Ultimately, cancer patients will get enormous benefits from such changes in clinical practice and very importantly, such changes will not incur significant cost on the healthcare system.

Our study is not without limitations. Firstly, leukaemia cells were pre-treated with propofol or sevoflurane before injection in *in vivo* models, which eliminates the complexities of drug metabolism *in vivo.* For further studies, it would be interesting to treat mice with propofol and sevoflurane directly. In addition, long-term studies could be established in which propofol and sevoflurane are given to leukaemia-bearing mice with established disease to mimic real clinical scenarios.

Secondly, the concentration of anaesthetics used for our study may be more applicable to paediatric patients. Clinical doses of general anaesthetics for invasive procedures in adult patients are generally lower and shorter in duration for such procedures. However, the clinical doses of general anaesthetics in paediatric patients are relatively high; for example, the use of 3.6% or higher concentrations of sevoflurane for paediatric leukaemia treatment procedures is well documented. ^3^^,^^26^^–^^28^ Additionally, the plasma concentration of propofol was reported to be high in paediatric procedures (plasma concentration range between 6 μg/ml to 2 μg/ml). ^3^ Furthermore, these patients are subject to frequent exposure to general anaesthetics. Therefore, the cumulative dose of anaesthetic exposure for this group of patients is considerably high.

Thirdly, isoflurane is used during the mouse study to provide long-term pain relief. It could be speculated that isoflurane may have some systemic effect on mice and possibly on injected leukaemia cells. However, we deem the effect is minimal given that systemic delivered isoflurane may need longer exposure time and more importantly repeated administrations to reach concentrations to significantly affect the experiment results as injected leukaemia cells were pre-treated ex-vivo before injections. In addition, if isoflurane had any effect on injected leukaemia cells, then such effect would affect sevoflurane group and propofol uniformly. Nevertheless, it is interesting to study the long term effect of isoflurane in future study.

## Conclusion

In the present study, we investigated the effect of two commonly used general anaesthetics, propofol and sevoflurane, on ALL cell migration and homing exemplified by 1) the number of cells entering bone marrow and 2) the homing location in relation to the nearest endosteal surface. Our data suggest that both general anaesthetics reduced the homing and migration of ALL cells *in vitro* and *in vivo.* Our results indicated that general anaesthetics reduced the surface CXCR4 expression. In addition, the adhesion of leukaemia cells to thrombin cleaved osteopontin (OPN) was reduced by general anaesthetics. In addition, both anaesthetics sensitised leukaemia cells to Ara-C possibly through CXCR4 mediated mechanisms.

We focussed on changes driven by ex vivo exposure to propofol and sevoflurane, however it is possible that these drugs might also re-program the bone marrow to be a more hostile environment for ALL cells and thus, in turn reduce the overall malignancy of ALL and reduce chemoresistance of ALL. Therefore, both anaesthetics may be safely used in patients undergoing procedures during ALL treatments but this warrants further clinical studies.

## Consent for publication

Not applicable.

## Ethics

All procedures performed relating to animal experiments in this study were approved by the Animal Welfare and Ethical Review Body (AWERB), Imperial College London and the Home Office, United Kingdom with PPL number 70/8496.

## Authors’ contributions

J.C., D.Q.M. and C.L.C. conceived the project. C.L.C. developed the intravital microscopy (IVM) method. J.C. performed all experiments, data analysis and wrote the manuscript. S.G.A. and X.M.L. helped perform the IVM experiment and helped IVM data analysis. All authors read and approved the final submission.

## Data Availability

Zendo: General anaesthetics reduce acute lymphoblastic leukaemia malignancies in vitro and in vivo via CXCR4 and osteopontin mediated mechanisms, https://doi.org/10.5281/zenodo.17268337. ^29^ This project contains following underlying data:
- Author Checklist - Full.pdf - Cell adhesion assay.xlsx - Flow data_vNew.xlsx - IVM data.xlsx - Nc cells.avi - propofol cells.avi - sevoflurane cells.avi - sub Figures F1000 JC.dox - Chemoresistance data.xlsx Data are available under the terms of the Creative Commons Attribution 4.0 International license (CC-BY 4.0).
