# Supplementary material for: General anaesthetics reduce acute lymphoblastic leukaemia malignancies in vitro and in vivo via CXCR4 and osteopontin mediated mechanisms
Source: F1000Res. 2025 Oct 9;11:1491. Originally published 2022 Dec 12. [Version 3] doi: 10.12688/f1000research.125877.3 (PMC11128051; doi:10.12688/f1000research.125877.3)
Supplement: Peer review history

# Reviewer report: Reviewer response for version 2

**DOI:** 10.5256/f1000research.162284.r273944

**Published:** 2024-05-25

**Authors:** Victor H. Villar Cortes

**Affiliations:** 1 School of medicine, University of St Andrews, St Andrews, Scotland, UK

The manuscript entitled “General Anesthetics Reduce Acute Lymphoblastic Leukemia Malignancies In Vitro and In Vivo via a CXCR4 and Osteopontin Mediated Mechanism” by Cui Jiang et al. presents interesting findings that demonstrate the use of anesthetics can inhibit the migration and homing of leukemia cells both in vitro and in vivo. Additionally, the study shows that both propofol and sevoflurane can synergize with chemotherapy (Ara-C). Although these results are interesting and suggest potential for clinical application, the mechanistic link between the compounds and autophagy induction requires further clarification. Therefore, I recommend accepting this manuscript with minor revisions.

 Comments:

1. **Figure 1**: Ethidium iodide staining primarily assesses necrosis, which typically requires more time to become apparent. To more accurately claim that propofol and sevoflurane do not alter cell viability, methods such as cell counting or the CCK-8 assay would be more appropriate.
2. **Figure 5 (a-d)**: The experiments assess the effects of propofol, sevoflurane, Ara-C, and their combinations after 4 hours. Is the CCK-8 readout influenced by the metabolic effects of Ara-C? Alternative methods for assessing cell viability should be considered to ensure accuracy.
3. **Figure 5 (e, f)**: The authors claim that while propofol in combination with Ara-C induces autophagy, sevoflurane in combination with Ara-C does not. However, autophagy flux, for example using chloroquine, was not assessed in these experiments. Furthermore, the comparable effects of both combinations (propofol/Ara-C and sevoflurane/Ara-C) on cell survival suggest that their impact on autophagy might not be relevant for cell survival. Further investigation into the autophagy flux and its relevance to cell survival is necessary.

Is the work clearly and accurately presented and does it cite the current literature?

Yes

If applicable, is the statistical analysis and its interpretation appropriate?

Yes

Are all the source data underlying the results available to ensure full reproducibility?

Yes

Is the study design appropriate and is the work technically sound?

Partly

Are the conclusions drawn adequately supported by the results?

Partly

Are sufficient details of methods and analysis provided to allow replication by others?

Yes

Reviewer Expertise:

cancer metabolism, hepatocellular carcinoma, biomarkers, liver physiology

I confirm that I have read this submission and believe that I have an appropriate level of expertise to confirm that it is of an acceptable scientific standard, however I have significant reservations, as outlined above.

# Reviewer report: Reviewer response for version 2

**DOI:** 10.5256/f1000research.162284.r246077

**Published:** 2024-03-09

**Authors:** Paul M Maciocia, Jongwon Yoon

**Affiliations:** 1 Department of Haematology, Cancer Institute, University College London, London, UK; 2 Haematology, University College London, London, England, UK

The authors have addressed our comments.

Is the work clearly and accurately presented and does it cite the current literature?

Yes

If applicable, is the statistical analysis and its interpretation appropriate?

Yes

Are all the source data underlying the results available to ensure full reproducibility?

Yes

Is the study design appropriate and is the work technically sound?

Partly

Are the conclusions drawn adequately supported by the results?

Partly

Are sufficient details of methods and analysis provided to allow replication by others?

Yes

Reviewer Expertise:

Cellular immunotherapy, ALL, lymphoma.

We confirm that we have read this submission and believe that we have an appropriate level of expertise to confirm that it is of an acceptable scientific standard.

# Reviewer report: Reviewer response for version 1

**DOI:** 10.5256/f1000research.138230.r187198

**Published:** 2023-08-21

**Authors:** Paul M Maciocia, Jongwon Yoon

**Affiliations:** 1 Department of Haematology, Cancer Institute, University College London, London, UK; 2 Haematology, University College London, London, England, UK

This is an interesting yet somewhat incomplete article: as the authors note, general anaesthetics may have some potential anti-cancer properties. The authors investigated the effect of two commonly used anaesthetics, propofol and sevoflurane, in acute lymphoblastic leukaemia cells. Anaesthetics-treated ALL cells showed reduced migration and homing both in vitro and in vivo, and the authors suggested its mechanisms. This article is concise and well-written, and provides meaningful messages. I have only a few comments.

1. In Figure 4a, the authors tested the different concentrations of propofol and the different exposure time of sevoflurane. Is there a rationale for the propofol exposure time (ie. 6 hours)?
2. As the authors mentioned, the effects of general anaesthetic are thought to be reversible. How long does the anaesthetic effect last in ex vivo treated ALL cells? This is quite important for establishing if the phenomena reported here might have any clinical impact
3. The authors used isoflurane for anesthesia when imaging intravital microscopy. Is there any possibility that isoflurane interferes with propofol or sevoflurane?
4. In patients, the anaesthetics may affect the activity of normal cells as well. From the therapeutic point of view, do the authors think the reduced migration of ALL cells will outweigh the slowing of normal cell activity?
5. One cell line is not enough to draw conclusions – the authors need to test multiple cell lines and primary B-ALL samples. This is the most critical point to accept for publication.
6. Its not at all clear what the functional consequences of these findings might be (I still don’t really know if anaesthetics are potentially good or bad for patients from this study) and I would like to see this explored more, though appreciate this would be a much more in-depth paper.

And here are my suggestions for minor revision:

1. It is unclear when intravital imaging was performed after ALL injection.
2. To represent multiple statistical significance between groups, it would be better to draw a line between comparing groups and put an asterisk mark over the line than ## symbol.
3. Introduction – ‘radiotherapy’ does not refer to diagnostic radiological procedures, there are also numerous typos

Is the work clearly and accurately presented and does it cite the current literature?

Yes

If applicable, is the statistical analysis and its interpretation appropriate?

Yes

Are all the source data underlying the results available to ensure full reproducibility?

Yes

Is the study design appropriate and is the work technically sound?

Partly

Are the conclusions drawn adequately supported by the results?

Partly

Are sufficient details of methods and analysis provided to allow replication by others?

Yes

Reviewer Expertise:

Cellular immunotherapy, ALL, lymphoma.

We confirm that we have read this submission and believe that we have an appropriate level of expertise to confirm that it is of an acceptable scientific standard, however we have significant reservations, as outlined above.

# Response

**Published:** 2024-02-03

**Authors:** cui jiang

Thanks this reviewer's insighful comment here is our point to point reply: 

 In Figure 4a, the authors tested the different concentrations of propofol and the different exposure time of sevoflurane. Is there a rationale for the propofol exposure time (ie. 6 hours)?

*Reply: We did run a time course experiment with propofol at the beginning of the project i.e., 2 and 4 hours of propofol exposure, we only found 6 hours of propofol exposure to produce significant effect; hence 6 hours of propofol exposure is used*

**

 As the authors mentioned, the effects of general anaesthetic are thought to be reversible. How long does the anaesthetic effect last in ex vivo treated ALL cells? This is quite important for establishing if the phenomena reported here might have any clinical impact

*Reply: As we have updated in the figure 4, propofol’s effect seems to be short-lived given after 24 hours of recovery time, propofol stop having effect on cxcr4 expressions but sevoflurane seems to have potent reductive effect on the CXCR4 expression given after 24 hours of recovery time, CXCR4 expression is still reduced*

**

 The authors used isoflurane for anesthesia when imaging intravital microscopy. Is there any possibility that isoflurane interferes with propofol or sevoflurane?

*Reply: This point has been discussed in the discussion section as systemic delivered isoflurane may need longer exposure time and more importantly repeated administrations to reach concentrations to significantly affect the experiment results as injected leukaemia cells were pre-treated ex-vivo before injections. In addition, if isoflurane had any effect on injected leukaemia cells, then such effect would affect sevoflurane group and propofol uniformly*

 In patients, the anaesthetics may affect the activity of normal cells as well. From the therapeutic point of view, do the authors think the reduced migration of ALL cells will outweigh the slowing of normal cell activity?

*Reply: Some studies have been carried out by our lab and others. And we found general anaesthetics have minimal toxicity against normal tissue. However, no one has studied whether general anaesthetics can reduce or interrupt normal bone marrow activity i.e., haematopoiesis given CXCR4 is an important  receptor for such activity. With this being said, for ALL patients, killing of leukaemia cells and re-shape bone marrow niche to be more hostile to ALL cells would likely outweigh the effect of general anaesthetics on normal bone marrow activity*

 One cell line is not enough to draw conclusions – the authors need to test multiple cell lines and primary B-ALL samples. This is the most critical point to accept for publication.

*Reply: Reh data has been added in the manuscript per comment*

 Its not at all clear what the functional consequences of these findings might be (I still don’t really know if anaesthetics are potentially good or bad for patients from this study) and I would like to see this explored more, though appreciate this would be a much more in-depth paper.

*Reply:Chemoresistance data has been added in the manuscript to show the functional consequence of general anaesthetics * And here are my suggestions for minor revision: It is unclear when intravital imaging was performed after ALL injection.

*Reply: Updated in the method section*

**

 To represent multiple statistical significance between groups, it would be better to draw a line between comparing groups and put an asterisk mark over the line than ## symbol.

*Reply:This has been rectified in all figures*

**

 Introduction – ‘radiotherapy’ does not refer to diagnostic radiological procedures, there are also numerous typos

*Reply: Added texts in introduction to minimise confusion*

# Reviewer report: Reviewer response for version 1

**DOI:** 10.5256/f1000research.138230.r158793

**Published:** 2023-01-26

**Authors:** Koichi Yuki

**Affiliations:** 1 Department of Anaesthesia, Harvard Medical School, Boston, MA, USA; 2 Division of Cardiac Anesthesia, Department of Anesthesiology, Critical Care and Pain Medicine, Boston Children's Hospital, Boston, MA, USA

This is the study that investigated the role of commonly used anesthetics propofol and sevoflurane in ALL cells, considering these drugs are often administered to patients with ALL. Authors found that anesthetics attenuated recruitment of ALL cells *in vitro* and *in vivo*.

 A very interesting study by the group that heavily studies the role of anesthetics. I have one very simple comment. 

1. As authors mentioned in the introduction, patients with ALL often receive procedures. Those procedures are often short (< 2 hours). Would you mind to elaborate your finding based on the duration of exposure?
2. What is the pathological significance of ALL cells recruited back to the bone marrow? Does this have any implication in the disease process? Authors found that ALL cells migrated less to the bone marrow. Does this effect last?

Is the work clearly and accurately presented and does it cite the current literature?

Yes

If applicable, is the statistical analysis and its interpretation appropriate?

Yes

Are all the source data underlying the results available to ensure full reproducibility?

Yes

Is the study design appropriate and is the work technically sound?

Yes

Are the conclusions drawn adequately supported by the results?

Yes

Are sufficient details of methods and analysis provided to allow replication by others?

Yes

Reviewer Expertise:

Anesthesia research

I confirm that I have read this submission and believe that I have an appropriate level of expertise to confirm that it is of an acceptable scientific standard, however I have significant reservations, as outlined above.

# Response

**Published:** 2023-02-27

**Authors:** cui jiang

Thank this reviewer for their kind comments

 Response

**Question 1:**

 We did acknowledge the longer exposure time is a limitation of the study. We have put in the discussion section: “ *Clinical doses of general anaesthetics for invasive procedures in adult patients are generally lower and shorter in duration for such procedures.*”            

 6 hours exposure time was chosen because:

1. We wish to do a proof-of-concept studies on whether long exposure time would yield significant results before designing shorter time period experiments given the how expensive it is to run such experiments.
2. Although the initial half-life of propofol and sevoflurane is very short and patients recover from anaesthesia within minutes, the terminal half-life of propofol and sevoflurane can be theoretically long (4-7 hours for propofol reference: *Khurram Saleem Khan, FCAI FJFICMI, Ivan Hayes, FCAI FJFICMANZ, Donal J Buggy, MD MSc DME FRCPI FCAI FRCA, Pharmacology of anaesthetic agents I: intravenous anaesthetic agents, Continuing Education in Anaesthesia Critical Care & Pain, Volume 14, Issue 3, June 2014, Pages 100–105* and up to 20 hours for sevoflurane reference: *Clin Pharmacokinet 1999 Jan; 36 (1). Page 19). *Long terminal half life may hypothetically give time for general anaesthetics to act on leukaemia cells

With all being said, we did perform in vitro experiments measuring cellular parameters for other parallel studies e.g. protetin expressions and in vitro functional assays with shorter time period i.e. 2 hour; we could see significant cellular changes following 2 hours exposure time of propofol and sevoflurane; we may publish those results in the near future

**Question 2:**

 Recruitment of ALL to the bone marrow is the first step of the homing process which allow the disease to happen in this murine model – given these mice are not genetically modified to have ALL genes. Reduction of recruitment of ALL cells to bone marrow translates into reduction in homing hence lower possibility of disease-causing cells in bone marrow.
